# Chemical Composition and Toxicity of *Achillea millefolium* L. Essential Oil Against *Acrobasis advenella* (Lepidoptera, Pyralidae) Under Laboratory Conditions

**DOI:** 10.3390/molecules30091927

**Published:** 2025-04-26

**Authors:** Edyta Górska-Drabik, Katarzyna Golan, Cezary Sempruch, Grzegorz Chrzanowski, Michał P. Dybowski, Monika Poniewozik

**Affiliations:** 1 Department of Plant Protection, Faculty of Horticulture and Landscape Architecture, University of Life Sciences in Lublin, 7 Leszczynskiego St., 20-069 Lublin, Poland; 2 Institute of Biological Sciences, Faculty of Sciences, Siedlce University, 14 Bolesława Prusa St., 08-110 Siedlce, Poland; cezary.sempruch@uws.edu.pl; 3 Faculty of Biotechnology, Collegium Medicum, University of Rzeszow, 8B Zelwerowicza St., 35-601 Rzeszow, Poland; 4 Department of Chromatography, Institute of Chemical Sciences, Faculty of Chemistry, Maria Curie Sklodowska University in Lublin, 3 Maria-Curie Sklodowska Sq., 20-031 Lublin, Poland

**Keywords:** *Aronia melanocarpa*, mortality, settling inhibition index, repellency, fecundity, phytotoxicity

## Abstract

The solutions of essential oil (0.5%, 0.8%, and 1.0% *w*/*v*) derived from *Achillea millefoilum* (EOAM) were evaluated for their repellent and insecticidal activity toward *Acrobasis advenella*, as well as their effects on the activity of selected larval tissue enzymes. The chemical composition of the EOAM analyzed by GC-MS showed that the main compounds included β-pinene, chamazulene, eucalyptol, and β-caryophyllene. Selection tests showed that larvae avoided inflorescences treated with 0.8% and 1.0% EOAM concentrations. The mortality of larvae increased with concentration and time of exposure to the EO, and the highest mortality rate was observed after 72 h. In addition, the impact of EO was examined on the activity of catalase (CAT), polyphenol oxidase (PPO), peroxidase (POX), α- and β-glucosidases, and acetylcholinesterase (AChE), i.e., important antioxidants, detoxification, digestive, and nervous system enzymes. A significant increase in CAT activity was found in larvae treated with 0.8% and 1.0% EOAM after both 24 and 48 h. Initially, treating larvae with 0.5% EO decreased β-glucosidase activity while increasing α-glucosidase activity. Moreover, 0.8% EOAM inhibited the activity of POX. These findings suggest that EOAM can affect important biochemical processes within the tissues of *A. advenella* larvae, making it a promising agent for the protection of black chokeberry crops.

## 1. Introduction

Poland is a leader in the production of chokeberry (*Aronia melanocarpa* [Michx.] Elliot) fruit and an exporter of finished and semi-finished chokeberry products, i.e., fruit concentrate and frozen foods. The area of chokeberry cultivation in Poland exceeds 14,000 hectares, with annual harvests reaching up to 54,000 tons, which accounts for approximately 70% of global production [1]. Chokeberry fruits are recognized as “superfoods” due to their higher content of phenolic compounds than most other blackberries. Key biologically active components found in these fruits include anthocyanins, carotenoids, flavonoids, tannins, minerals (B, I, Mg, Cu, and Mo), and vitamins (C, B2, B6, E, P, and PP) [2].

Previously, *A. melanocarpa* was considered a reliable crop, primarily due to its low environmental requirements and low human intervention in pest and disease control. Plantation management was mainly limited to maintenance practices and fertilization and did not require the use of insecticides and fungicides [3]. Over the past two decades, the situation has changed, and chokeberry producers are now forced to use chemical plant protection agents, primarily to control *Acrobasis advenella* caterpillars (Lepidoptera: Pytalidae, Phycitine) [4,5]. *A. advenella* is widely distributed throughout Europe. This oligophagous species primarily feeds on plants from the family *Rosaceae*, particularly those from the genera *Sorbus* L. and *Crataegus* L. [6]. Early spring feeding by caterpillars on buds leads to a reduction in yield (on average by 20%) [7] and triggers a follow-up response, resulting in decreased levels of sugars, total flavonoids, and anthocyanins in the fruit. The quality of the raw material is also adversely affected by the feeding of newly hatched caterpillars on immature chokeberries [5]. The adaptation of this species to a new host plant and its widespread occurrence on chokeberry plantations in Poland have made it nearly impossible to achieve both high-quality and satisfactory yields without black chokeberry comprehensive *A. advenella* control [8]. Chemical insecticides are used to prevent the negative effects of pest feeding on chokeberry buds and fruits. However, these insecticides can impact both the environment and the quality of the fruit [9]. Pyrethroid-based products, such as deltamethrin and lambda-cyhalothrin, are commonly used agents. However, many pyrethroids are known to be toxic to mammals, aquatic organisms, and other wildlife [10,11]. They also contribute to groundwater pollution and disrupt agricultural ecosystems [12,13]. Considering the health benefits of chokeberries and their applications in the pharmaceutical [14] and cosmetic industries [15], it is preferable that the plant material is sourced from organic plantations, free from pesticide use.

An environmentally safe alternative is to replace chemical products with natural substances, such as essential oils (EOs) [16]. As natural compounds, EOs are part of modern trends in plant protection, particularly in the context of organic farming. EOs are well known for their antibacterial, antifungal, antimite, and overall insecticidal properties [17,18,19,20,21]. Their effectiveness is the result of complex interactions between the insect and the host plant [22]. EOs can disrupt the identification of host plants, inhibit egg-laying, and negatively affect the growth and development of insects [23,24,25]. These compounds have documented biocidal, repellent, and antifeedant properties [26,27].

EOs, as aromatic oily extracts, have been one of the most popular alternatives to synthetic chemical insecticides for pest management. EOs show a wide range of effects against insects, acting as repellents, attractants, or antifeedants, or inhibiting respiration and oviposition, impeding host plant identification, and reducing adult emergence by ovicidal and larvicidal activity [23,25,28,29,30,31]. However, the effect of EOs depends strongly on their composition, which is largely determined by genetic variation and environmental conditions [32,33,34].

In recent years, there has been a marked increase in interest in products based on natural plant compounds. This trend may be caused by the gradual withdrawal of active substances used in chemical plant protection products, which are now considered harmful to humans and dangerous to the environment [35]. There is also a noticeable increase in the number of organic farms, driven by the demand for natural raw materials free from pesticide residues [36]. This is the case with black chokeberry, whose fruits from such plantations are sought after for their superior quality, particularly for use in the pharmaceutical and cosmetic industries [2,37].

*Achillea* species from the family Asteraceae are globally recognized medicinal herbs and have been the focus of numerous pharmacological studies [38,39]. *A. millefolium* L. shows significant pharmacological activity and has emerged as the most prominent species within the genus [40,41]. The EO of *A. millefolium* (EOAM) exhibits a broad spectrum of biological activities, including insecticidal properties [42]. The negative (toxic, repellent, antifeedant) effects of EOAM have been documented by many researchers for representatives of various groups of harmful insects: beetles (Coleoptera) [43,44], moth caterpillars (Lepidoptera) [45,46], aphids (Hemiptera, Aphididae) [42,47], and mites (Acari) [48].

The EO can negatively affect different functional systems in insects, usually due to the synergistic interactions that occur in single, natural, and complex mixtures, acting by reducing the resistance of these organisms [49,50]. It is also well known that insects use detoxification enzymes to metabolize deleterious secondary plant metabolites. Antioxidant insect enzymes, such as catalase (CAT), polyphenol oxidase (PPO), and peroxidase (POX), participate in the detoxification of reactive oxygen species (ROS) generated in response to plant allelochemicals, such as phenolics or quinines [51,52,53]. Additionally, various secondary metabolites can affect the digestive processes of insects by altering enzyme activities [54]. Importantly, the reactions are particular and different depending on the EO used and the targeted insect species [55].

While EOAM exhibits a broad range of biological activities, its toxicity, impact on *A. advenella* biological parameters, and effect on the functioning of enzymatic systems have not been elucidated. Our previous studies on the effect of plant extracts and EOs on the behavior of larvae and females of *A. advenella* showed that 0.1% EOAM was one of three EOs (besides *Tanacetum vulgare* and *Satureja hortensis*) exhibiting repellent properties against larvae and females during the egg-laying period [56].

The main purpose of this study was to determine the effect of various concentrations of *A. millefolium* essential oil (EOAM) (0.5, 0.8, and 1.0%) on larval mortality, pupation, emergence percentage, longevity, and fertility of *A. advenella*. Moreover, our research hypothesis posited that the analyzed concentrations of EOAM would disturb the functioning of antioxidant, detoxifying, digestive, and nervous enzymatic systems through changes in the activity of crucial enzymes such as catalase (CAT), peroxidase (POX), polyphenol oxidase (PPO), α- and β-glucosidases, and acetylcholinesterase (AChE). Moreover, the repellent properties of various concentrations of EOAM against *A. advenella* larvae have been investigated, as well as its potential phytotoxicity in relation to the oil’s chemical composition.

## 2. Results

### 2.1. EOAM Chemical Composition

**Figure 1:**
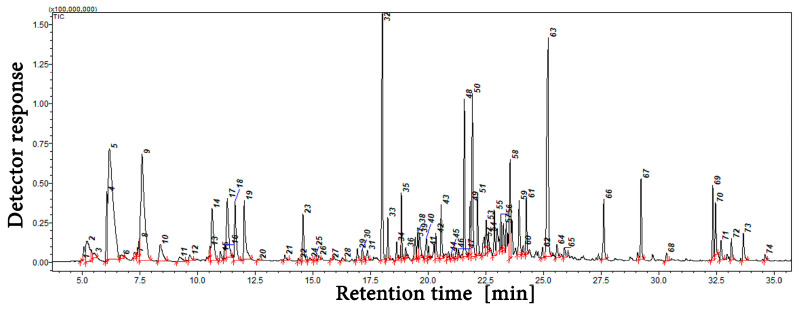
GC-MS chromatogram of the essential oil isolated from *Achillea millefolium* L.

The results of the qualitative and quantitative analysis of *A. millefolium* EO are presented in Table 1 and Figure 1 (gas chromatogram) and Supplementary Material (MS spectra for unidentified substances). A total of 66 compounds were identified, representing 81.07% of the EO content. The primary compounds included β-pinene (12.84%), eucalyptol (9.15%), chamazulene (9.05%), and β-caryophyllene (7.26%). The percentage of the remaining 62 constituents ranged from 4.47% to 0.06%.

### 2.2. Settling Inhibitory Activity (SI)

The free-choice bioassays showed that fewer *A. advenella* larvae settled on inflorescences treated with 0.8% and 1.0% EOAM than on the controls. The SI values ranged from 37.5% to 45.3% (Table 2).

### 2.3. EOAM Effect on the Mortality of A. advenella larvae

**Figure 2:**
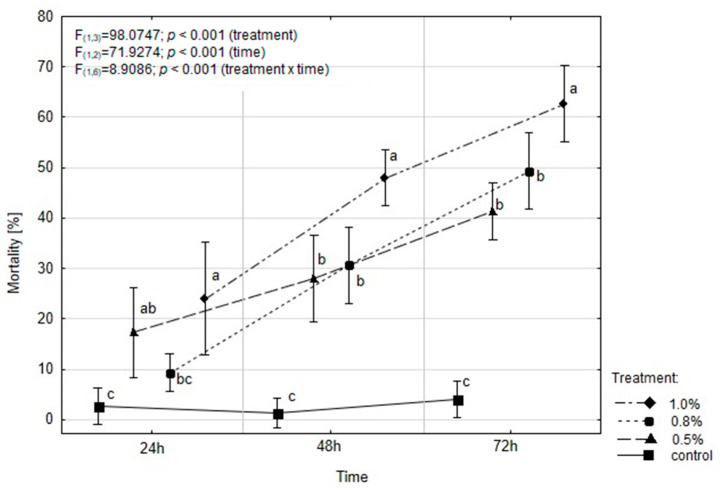
Effect of EOAM on mortality (%) of *Acrobasis advenella* larvae. The data in the figure are mean ± SD (standard deviation) of three replicates. Points marked by different letters are significantly different (separately for each time period) (Tukey’s HSD test *p* ≤ 0.05).

The mortality rate of *A. advenella* larvae was significantly affected by both the treatment and duration of exposure. Additionally, a significant interaction between treatment and time was observed (Figure 2). After 24 h, dead caterpillars were found in all experimental variants, including the control. The highest mortality rate (24.0%) was observed for 1% oil concentration, and this value doubled after 48 h. A similar proportion of dead caterpillars (49.33%) was recorded for 0.8% oil, but only after 72 h. The application of 1% EOAM resulted in a caterpillar mortality rate of 62.66%. Statistical analysis revealed that all tested concentrations of EOAM significantly affected caterpillar mortality after 48 and 72 h compared to the control.

The estimated lethal concentration values (LC_50_) with corresponding confidence limits (95%) after 24, 48, and 72 h of exposure to the tested concentrations of EOAM are shown in Table 3. Only the 1.0% concentration showed significant insecticidal properties against *A. advenella* larvae after 72 h of exposure (LC_50_ = 0.99%). The LC_50_ values were significantly different between 24 and 72 h exposure times, as evidenced by the non-overlapping confidence intervals.

### 2.4. EOAM Effects on A. advenella Demographic Parameters

All concentrations of EOAM tested in the experiment significantly influenced the process of pupation compared to the control, where 93.33% of caterpillars successfully emerged as adults. The lowest number of pupae was observed for 1% EOAM, with only 15.55% of caterpillars pupating (Table 4). However, statistical analysis did not reveal any effect of the tested EOAM concentrations on other demographic parameters of *A. advenella*, such as adult emergence, longevity, and fertility (Table 4).

### 2.5. EOAM Phytotoxicity

A thorough examination of sprayed shoots did not reveal any toxic effects on black chokeberry plants sprayed with EOAM solutions. Exposure time did not influence the severity of phytotoxicity. Even at the highest tested concentration (1.0%), the EO did not cause any plant damage. Both 48 and 72 h after application, the percentage of leaf area showing symptoms did not exceed 1% (Table 5).

### 2.6. EOAM Effect on Larval Enzymes

**Figure 3:**
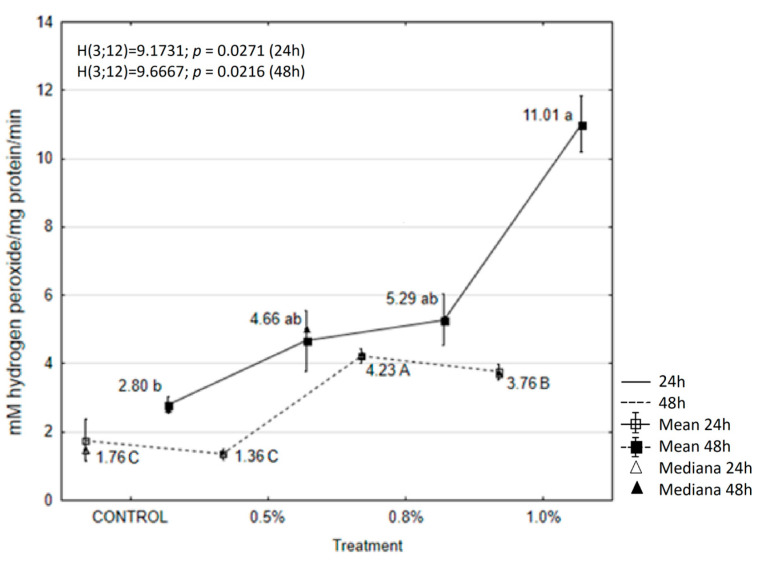
The effects of EOAM at different concentrations on catalase (CAT) in *Acrobasis advenella* larvae. Results are reported as mean ± SD (standard deviation), calculated from three replicates. Points marked by different letters are significantly different (Kruskal–Wallis, Dunn’s test, *p* ≤ 0.05).

**Figure 4:**
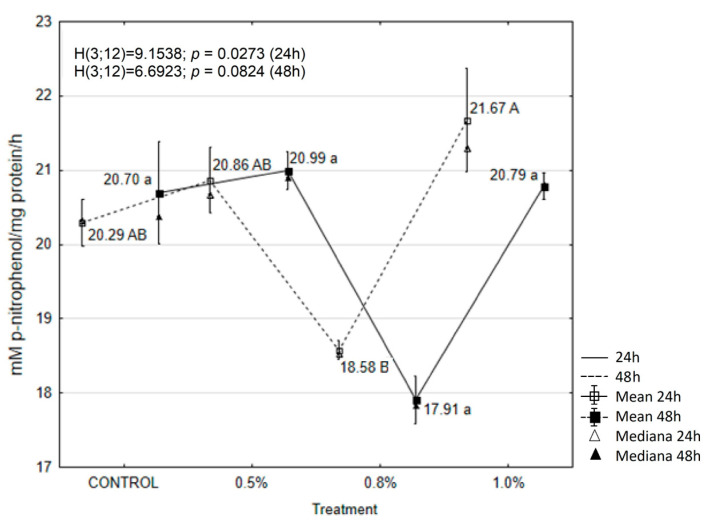
The effects of EOAM at different concentrations on peroxidase (POX) in *Acrobasis advenella* larvae. Results are reported as mean ± SD (standard deviation), calculated from three replicates. Points marked by different letters are significantly different (Kruskal–Wallis, Dunn’s test, *p* ≤ 0.05).

**Figure 5:**
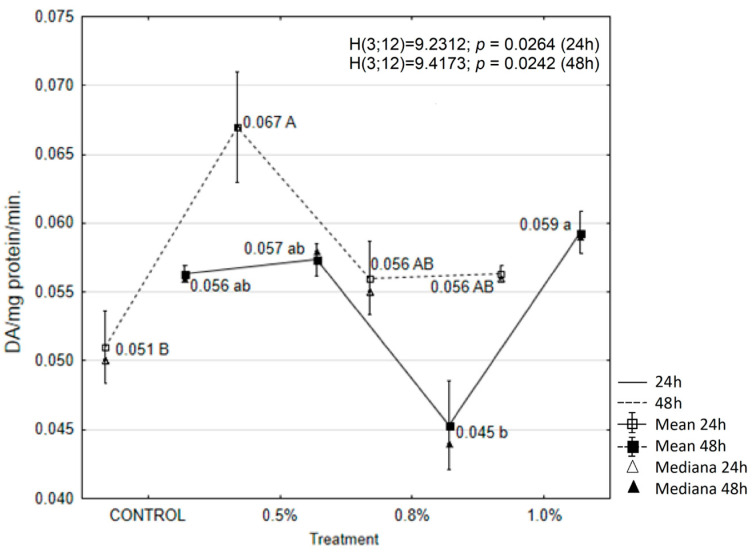
The effects of EOAM at different concentrations on polyphenol oxidase (PPO) in *Acrobasis advenella* larvae. Results are reported as mean ± SD (standard deviation), calculated from three replicates. Points marked by different letters are significantly different (Kruskal–Wallis, Dunn’s test, *p* ≤ 0.05).

**Figure 6:**
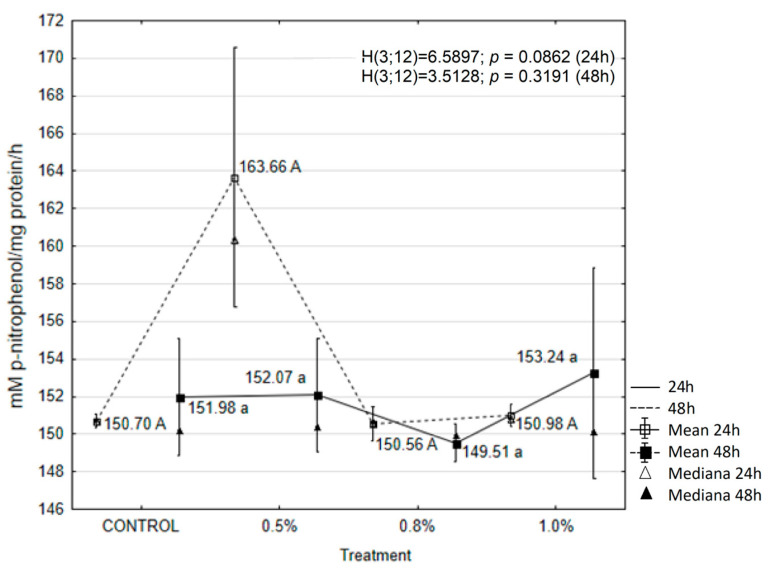
The effects of EOAM at different concentrations on α-glucosidase in *Acrobasis advenella* larvae. Results are reported as mean ± SD (standard deviation), calculated from three replicates. Points marked by different letters are significantly different (Kruskal–Wallis, Dunn’s test, *p* ≤ 0.05).

**Figure 7:**
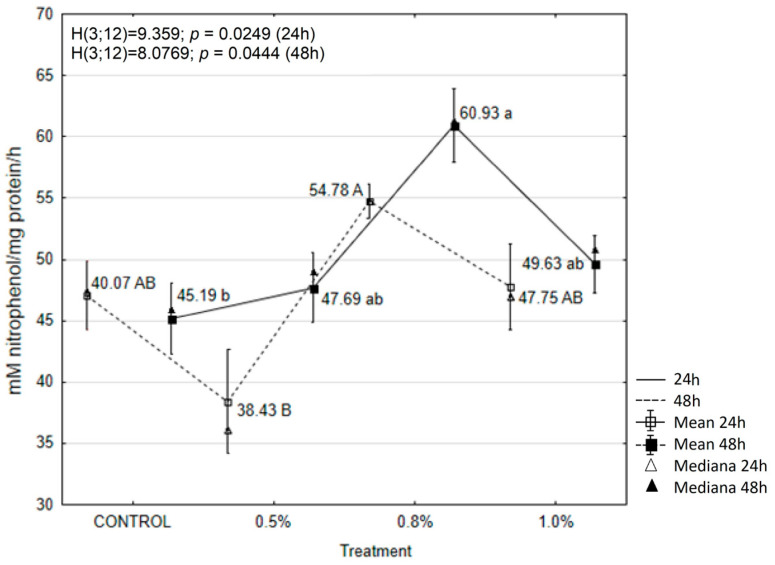
The effects of EOAM at different concentrations on β-glucosidase in *Acrobasis advenella* larvae. Results are reported as mean ± SD (standard deviation), calculated from three replicates. Points marked by different letters are significantly different (Kruskal–Wallis, Dunn’s test, *p* ≤ 0.05).

**Figure 8:**
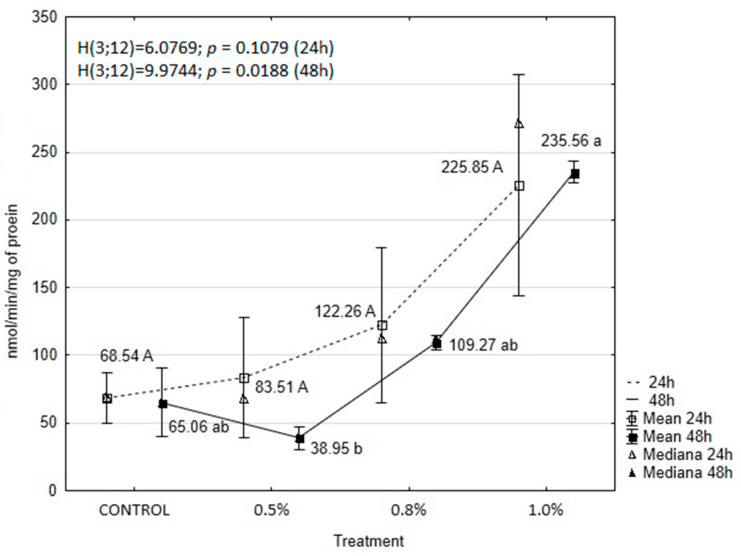
The effects of EOAM at different concentrations on acetylcholinesterase (AChE) in *Acrobasis advenella* larvae. Results are reported as mean ± SD (standard deviation), calculated from three replicates. Points marked by different letters are significantly different (Kruskal–Wallis, Dunn’s test, *p* ≤ 0.05).

The results showed that treatment with the tested concentrations of EOAM significantly affected the activity of enzymes in the tissues of *A. advenella* larvae, with the exception of peroxidase (POX) and α-glucosidase. Enzyme activities changed depending on the treatment and exposure time (Figure 3, Figure 4, Figure 5, Figure 6, Figure 7 and Figure 8).

The activity of catalase increased after oil application in all experimental periods. A significant increase in catalase activity was observed for 0.8% and 1.0% EOAM after 24 h, with activity levels reaching 2.40 and 2.14 units, respectively (Figure 3). Overall, a stronger induction was observed 48 h after treatment with 1.0% EOAM, when the activity of this enzyme increased five-fold compared to the control (Figure 3).

Conversely, POX activity decreased significantly after the application of 0.8% EOAM at both 24 and 48 h. However, these changes were not statistically significant compared to the control (Figure 4).

The activity of polyphenol oxidase (PPO) increased during the first experimental period following the application of all tested EOAM concentrations compared to the control (Figure 5). The highest and statistically significant increase in PPO activity occurred 24 h after the application of 0.5% EOAM.

Treatment of *A. advenella* larvae with 0.5% EOAM resulted in an increase in α-glucosidase activity after 24 h, although these changes were not significantly different (Figure 6). There were also no statistically significant changes in the activity of this enzyme after 48 h.

Regarding β-glucosidase activity, no statistically significant differences were observed 24 h after treatment with any of the tested EOAM concentrations. Nevertheless, a significant increase (1.35 units) in β-glucosidase activity was recorded 48 h after the application of 0.8% EOAM compared to the control (Figure 7).

EOAM treatment also affected the activity of acetylcholinesterase in the tissues of *A. advenella* larvae. The activity of this enzyme varied with both treatment and time (Figure 8). AChE activity significantly increased after treatment with all tested concentrations throughout the experimental periods, except for 0.5% EOAM at 48 h compared to the control. Among the tested oil concentrations, the strongest effect was observed after applying 1.0% EOAM, with AChE activity showing 3.3-fold and 3.6-fold increases at the respective time points compared to the control. Despite these marked changes in enzyme activity, statistical analysis did not reveal significant differences compared to the control.

## 3. Discussion

It is well established that the composition of EOs is highly variable, and the content of individual EO components depends not only on the origin of the plant material but also on many other factors, e.g., harvest season, fertilization, extraction method, or plant tissue [22,57,58,59,60]. Previous studies have documented the chemical compositions of *A. millefolium* EOs from various regions across different countries and continents [42,44,48,60,61,62]. In Poland, research has also indicated variability in both the quantity and composition of the main chemical compounds found in *A. millefolium* EO. Zawiślak and Nurzyńska-Wierdak [63] reported 95 components in their analyses of the EO from this species. However, the present study identified 29 fewer compounds than those reported by the aforementioned authors. Interestingly, Czerniewicz et al. [42] and Bączek et al. [64] identified even fewer compounds in EOAM compared to the number of components determined in the present work (39 and 40 fewer, respectively).

As shown in the literature, different chemotypes of *A. millefolium* EO have been identified, including sabinene-type oils [65], 1,8-cineole [66], β-pinene [67,68,69], chamazulene, and p-cymene [70,71]. Judzentiene and Mockute [61] demonstrated significant differences in the chemical composition of EO originating from different *A. millefolium* forms. For example, the β-pinene content varied from 0.5% to 20.0% in plants within the same populations. The chemical composition of EOAM detected in this study is consistent with previous findings from Poland. Bączek et al. [64] identified three chemotypes of *A. millefolium* oil: β-pinene, β-pinene + chamazulene, and 1,8-cineole. The EOAM in this study, characterized by a β-pinene + chamazulene chemotype, had a similar chemical profile to that described by Zawiślak and Nurzyńska-Wierdak [63]. The main constituents of the latter EO were β-pinene and chamazulene, both present in higher percentages (respectively, 19.32% and 14.41%) than previously reported. This oil also contained caryophyllene (E-isomer), although eucalyptol was not isolated. On the other hand, a slightly different composition of *A. millefolium* EO was reported by Czerniewicz et al. [42], where of 27 identified compounds, chamazulene was dominant, and β-pinene was present in lower quantity. Intra-specific genetic divergence may also account for this chemical variability [61].

The present results demonstrated that EOAM effectively inhibited *A. advenella* larvae from settling on treated inflorescences, with the level of inhibition varying depending on the concentration applied. The highest activity was observed at concentrations of 0.8 and 1.0% EOAM. Our previous research demonstrated the repellent properties of plant extracts and EOs from eight different plant species on the behavior of *A. advenella* caterpillars and females. Repellent properties have already been demonstrated at a concentration of 0.1% EOAM [56].

The repellent properties of EOAM have been confirmed by many literature reports. For instance, Tampe et al. [44] demonstrated the effectiveness of EOAM in repelling *Aegorhinus nodipennis* (Hope) (Coleoptera: Curculionidae); additionally, the mixture of metabolites present in EOAM disrupted physiological processes and exhibited neurotoxic effects. These authors highlighted the potential of EO, particularly those containing α- and β-thujone isomers, as effective repellent agents. Their analysis identified the presence of 11 compounds, with β-thujone being the dominant one, accounting for 96.2% of the total content. The toxic properties of β-thujone on insects have been well documented in numerous studies [56,72,73,74]. Additionally, other EOAM components, such as β-pinene, chamazulene, β-caryophyllene, and eucalyptol, identified in our experiments, also demonstrated insect-repellent properties. The repellent potential of β-pinene against *Panaphis juglandis* (Goeze) and *Chromaphis juglandicola* (Kalt.) aphid larvae (Hemiptera; Aphidoidea; Drepanosiphinae) has been documented by Krzyżanowski [75]. Meanwhile, Czerniewicz et al. [42] demonstrated the repellent and biocidal properties of EOAM against *Myzus persicae* (Sulzer). However, the EOAM examined by the aforementioned authors differed from the one tested in the present study, as the 1,8-cineole (9.2%) was the dominant component in that oil; however, β-pinene was also present in a high proportion (6.6%). A study by Conti et al. [69] also confirmed the larvicidal activity of EOAM against the mosquito *Aedes albopictus* (Skuse) (Diptera: Culicidae), identifying eucalyptol (14.2%) and β-pinene (12.4%) as the main components, which were also dominant in our analysis. Similarly, Nenaah [43] reported EOAM fumigant toxicity against *Tribolium castaneum* (Herbst) (Coleoptera, Tenebrionidae), with chamazulene (26.2%) and β-pinene (16.6%) being the primary constituents. The author showed a weak effect of EOAM on both the reduction of adult abundance and demographic parameters of this species (larval and pupal development, number of individuals reaching successful adult emergence, total life span, and sex ratio). It should be noted that the toxicity of EOs significantly increased when they were formulated as nano-emulsions.

The repellent and biocidal properties of EOAM have also been demonstrated against other insect species, e.g., *Rhopalosiphum maidis* (Fitch) (Hemiptera: Aphididae) [47] and *Plodia interpunctella* (Hübner) (Lepidoptera: Pyralidae) [76]. Halbert et al. [47] conducted an experiment involving 55 substances, including 1.0% oil from *A. millefolium*, to assess their repellent effects on aphids. The results of the experiment demonstrated a high repellent potential of *A. millefolium* oil compared to the other substances tested. The mean number of aphids on treated and untreated plates was −1.72 and 3.94 at *p* = 0.0041, respectively. On the other hand, Ebadollahi and Ashouri [76] showed a strong fumigant activity of EOAM against adults of *P. interpunctella* (100% mortality when the adults were exposed to 65 μL/l concentrations of *A. millefolium* after 48 h). However, these authors did not determine the chemical profile of the *A. millefolium* EOs tested in their study.

Our study showed that caterpillar mortality increased over time, with significant mortality observed only after 72 h (LC_50_ = 0.99%). In our previous works using 0.5% oils from *T. vulgare* and *S. hortensis* against *A. advenella* larvae, the effects were observed only 72 h after application [28,29]. *S. hortensis* EO was significantly less effective, with nearly 38% larval mortality after 72 h, compared to *T. vulgare* EO and the EOAM tested in this study. The varying toxicity of these three EOs in contact with *A. advenella* larvae suggests a difference in the sensitivity of larvae of this pest to individual EOs, which could be attributed to variations in the molecular weights of individual compounds and their capacity to penetrate larval tissues [77]. Czerniewicz et al. [42] reported a stronger effect of EOAM on *M. persicae* as early as 24 h (LC_50_ = 0.34%) and 48 h (LC_50_ = 0.30%) after treatment, with significant inhibition of aphids’ enzymatic activity also observed during this period. Ebadollahi and Ashouri [76] found that EOAM application resulted in 100% mortality of *P. interpunctella* within 48 h after treatment, confirming its high potential as a fumigant against stored-product pests. Our research, on the other hand, showed that a significantly higher concentration of EOAM (1.85%) would have to be applied to achieve 50% mortality of caterpillars after 24 h. Naghizadeh et al. [46] also evaluated the effect of EOAM on *Phthorimaea operculella* Zeller (Lepidoptera: Gelechiidae), showing that treating the food source (potato tubers) with a 0.5% oil concentration reduced egg-laying and shortened insect lifespans. Moreover, sublethal concentrations of *A. millefolium* oil affected a key pest control parameter, namely the sex ratio, leading to a higher proportion of males in the population.

Since the chemical components of EOs are lipophilic, they can penetrate insect tissues and influence enzyme activity [78]. We stated that the effect of EOAM on *A. advenella* was connected with the induction of the activity of most of the analyzed enzymes, except for POX and α-glucosidase. The increase in enzymatic activity was usually induced by the higher of the tested concentrations (0.8 or/and 1.0%), with the exception of PPO, whose activity increased after 24 h of exposure to EOAM at 0.5% concentration. In the case of AChE and CAT, the increase in activity occurred after 24 h of exposure to the tested preparations and was maintained in the latter, while in the case of β-glucosidase, this effect was observed only after 48 h. The obtained data partially confirm the results of previous studies, which found that *S. hortensis* EOs induced PPO and α- and β-glucosidase activity in the tissues of *A. advenella* larvae, while EOs from *T. vulgare* induced an increase in CAT and PPO activity in the same species, while inhibiting POX and α- and β-glucosidase [28,29]. Our results and previous data indicate that EOs produced by different plant species are characterized by various chemical compositions and, therefore, may differentially modify insect physiology. Their composition is dominated by one to several components, which often have similar insecticidal activity as the entire mixture. It is widely believed that qualitative and quantitative changes in AChE are responsible for insect resistance to insects. Fournier et al. [79] reported that in *Drosophilla melanogaster* (Meigen) strains producing different amounts of AChE via P-mediated transformation, resistance to organophosphate pesticides correlates with the amount of the enzyme in the central nervous system. Inhibition of this enzyme disrupts the coordination of the neuromuscular system, finally leading to paralysis and death of the insect. Czerniewicz et al. [42] found that EOs from Asteraceae plants inhibited AChE activity in tissues of female *M. persicae*, and their concentration correlated with aphicidal potential. Similar activity was also observed for β-phellandrene (the main component of Santolina EOs) against *Reticulitermes speratus* (Kolbe) and 1,8-cineole (the main component of yarrow and tansy oils) against *Aedes aegypthi* (L.) [80,81]. It is therefore possible that the increase in AChE activity in *A. advenella* larvae treated with 0.8 and 1.0% EOAM concentrations indicates a high tolerance of this species to the tested substances. Induction of CAT and PPO activities in *A. advenella* tissues suggests that the response is at last connected with oxidative stress. Cytotoxic properties of EOs may result from the presence of phenolics, aldehydes, and alcohols in their composition [82]. These biomolecules may affect the fluidity of the membranes, causing the building of radicals, cytochrome C, calcium ions, and proteins as a result of oxidative stress and bioenergetic failure. Forty-eight-hour exposure of the larvae to EOAM also resulted in an increase in β-glucosidase activity. A similar effect in relation to the tested insect species was previously observed in the case of *S. hortensis* EOs and their main component carvacrol. These substances induced a 194.08% and 214.67% increase in β-glucosidase activity in larval tissues, respectively [28]. On the other hand, Magierowicz et al. [56] and Czerniewicz et al. [83] observed inhibition of this enzyme following treatment of *A. advenella* with *T. vulgare* EOs and *Rhopalosiphum padi* (L.) with EOs from Asteraceae plants. Changes in β-glucosidase activity could affect the metabolism of sugars and osmoregulation in *A. advenella* tissues due to their participation in the hydrolytic decomposition of some oligosaccharides. On the other hand, this enzyme may participate in plant defense reactions by releasing toxic aglycones from less harmful glycosides [83].

Nevertheless, the phytotoxic properties of EOs have long been a major drawback to their potential broader application, as they can have detrimental effects on cultivated plants [84]. In the present study, EOAM rich in β-pinene and eucalyptol showed no phytotoxicity to *A. melanocarpa* leaves even when administered at the highest concentrations tested. Neither the exposure time nor the concentration of EOAM had a significant impact on *A. melanocarpa* leaves and shoots. Both the lowest and highest spray concentrations caused phytotoxic symptoms in less than 1% of the leaf area. Czerniewicz et al. [42] reported different outcomes, where the highest concentration (0.8%) tested led to the development of phytotoxic symptoms on more than 50% of pea leaf surfaces. This discrepancy was likely due to differences in the anatomical structure of the leaves between the two plant species.

## 4. Materials and Methods

### 4.1. Insect Culture and Rearing Conditions

The fourth-stage (L_4_) of *A. advenella* larvae were collected in May with inflorescences from an organic horticulture farm of black chokeberry in Samoklęski (51.4500 °N, 22.4333 °E) located in southeastern Poland, Lublin Province. Subsequently, they were reared in laboratory conditions at the University of Life Sciences in Lublin (Poland). The larvae (*n* = 795) were kept separately in Petri dishes (9 cm in diameter) and fed with *A. melanocarpa* inflorescences. Petri dishes were cleaned, and fresh inflorescences (each approx. 0.7 g) were provided daily. The larvae were used for toxicity tests, as well as demographic and biochemical experiments.

### 4.2. Plant Material and EOAM Extraction

Dry *A. millefolium* aerial parts were purchased as an organic product (PL-ECO-01) from Dary Natury Co. (Grodzisk, Poland). For the extraction process, 100 g of plant material was placed in a 1000 mL round-bottom flask with 500 mL of distilled water and subjected to hydrodistillation for 4 h using a Clevenger apparatus. The water extracts of EOs were dried over anhydrous sodium sulfate and stored in amber vials at 4 °C in the dark until GC-MS analysis [85]. The EO yield (expressed as a percentage) was calculated as the ratio of the weight of extracted oil (0.31 g) to the weight of plant material.

### 4.3. Compound Identification and Estimation by Gas Chromatography–Mass Spectrophotometry

Qualitative analysis of EOs was performed using a GC/MS QP2010 system (Schimadzu, Kyoto, Japan) equipped with a ZB5-MSi fused silica capillary column (30 m × 0.25 mm id. 0.25 μm film thickness, Phenomenex). Helium (grade 5.0) was used as the carrier gas, with a flow rate of 1 mL/min. The injection temperature was set at 310 °C, and 1 μL of the sample was injected in split mode (purge time: 0.7 min). The following temperature program was applied: initial hold at 50 °C for 2 min, followed by a linear temperature increase of 5 °C/min until reaching 310 °C. The mass spectrometer operated in EI mode at 70 eV, with the ion source temperature set at 220 °C. The mass range scanned was from 35 to 450 amu. Qualitative analysis was performed by comparing the retention indices and mass spectra for the obtained peaks with corresponding data from the NIST’14 mass spectrometry library. Quantitative analyses were carried out using a gas chromatograph with a GC/FID GC2010 flame ionization detector (Schimadzu, Kyoto, Japan). Hydrogen was used as the carrier gas at a flow rate of 1 mL/min. The experimental conditions were the same as for GC/MS. Peak identification was performed based on experimentally determined retention indices [86,87].

### 4.4. Laboratory Bioassays

All bioassays were performed at 22 ± 1 °C, 70 ± 5% relative humidity, and a 16:8 h photoperiod. EOAM concentrations of 0.5%, 0.8%, and 1.0% (*w*/*v*) were used for all tests, including demographic and biochemical analyses. The 0.5% concentration (*w*/*v*) was prepared by mixing 0.5 mL of EOAM with 2% (*v*/*v*) ethanol (99.8%), distilled water, and 0.5 mg of Tween 80 as an emulsifier. The remaining concentrations were prepared in a similar manner, adjusting the volume of EOAM to 0.8 mL for the 0.8% concentration and 1 mL for the 1.0% concentration. A mixture with the same composition but without the EO was used as a control solution [56].

#### 4.4.1. Settling Inhibition Bioassays

The potential of EOAM at concentrations of 0.5%, 0.8%, and 1.0% (*w*/*v*) against colonization of *A. advenella* larvae was evaluated using free-choice tests. Initially, *A. melanocarpa* inflorescences were immersed in EOAM or control solution for 15 s and dried at room temperature for 15 min. The dried inflorescences were positioned at the periphery of Petri dishes (20 cm diameter) lined with moistened filter paper. Each dish contained four inflorescences treated with EOAM and four untreated control inflorescences. Next, 15 larvae were released in the center of each dish, and after 24 h, the number of insects that settled on each inflorescence was recorded. Each concentration of EO was tested in triplicate. The settling inhibition index (%SI) was calculated as follows: %SI = [1 − (%T/%C)] × 100, where %T is the percentage of larvae on EO-treated inflorescences, and %C is the percentage of larvae on control inflorescences [88].

#### 4.4.2. EOAM Toxicity Test on Larvae

The toxicity assay involved spraying larvae of the same age and black chokeberry inflorescences with the selected EOAM concentrations (*w*/*v*) or a control solution. Each treatment was sprayed three times using a 100 cm³ manual plastic sprayer (Puro Cosmetics, Wrocław, Poland). The sprayer’s contents were vigorously mixed before application, with approximately 0.40 cm³ of solution used per treatment performed in triplicate. Each Petri dish contained a single larva [28]. Mortality was recorded at 24, 48, and 72 h after exposure to the EOAM, and LC_50_ values were calculated using Probit analysis [89]. Larvae were considered dead when they did not respond to stimulation with a camel-hair brush. Mortalities observed in the treatment groups were corrected for natural mortality recorded in the control group. The experiments were conducted using 15 larvae per concentration of EOAM and the control, with five replicates per treatment.

#### 4.4.3. EOAM Influence on Demographic Parameters

Three tested concentrations (0.5%, 0.8%, and 1.0% *w*/*v*) were used to evaluate the effects of EO on the population parameters of *A. advenella*. The experiment was conducted in Petri dishes, where larvae and chokeberry inflorescences were sprayed with the tested EOAM concentrations. After 3–4 days, soil was added to each Petri dish to create suitable conditions for pupation. Daily observations were made, with soil moisture maintained using an atomizer. When the larvae were no longer present on the host plant, the soil was inspected to determine the exact moment the larvae descended for pupation. The study recorded the number of pupae, emerged moths, their longevity, and oviposition of *A. advenella*. Longevity results were expressed as minimum, maximum, and mean percentages. The percentages of pupation and adult emergence were calculated as follows: pupation (%) = 100 × total number of pupae formed/total number of larvae released and adult emergence (%) = 100 × total number of adults formed/total number of pupae. Each concentration of EOAM, as well as the control, was tested on 15 larvae six times [56].

After the moths emerged, their sex was determined, and then they were paired and placed in rearing cages (31 cm × 21 cm × 21 cm) (TRIXIE Co, Kobylarnia, Poland). Immature infructescences (egg-laying sites) were placed in floral foam to preserve their freshness. One pair of newly emerged *A. advenella* adults (male and female) were released into each cage. The moths were fed with a 20% honey solution. Immature fruits were checked every day, recording the number of eggs laid on each infructescence. The experiment included 40 pairs of moths (10 pairs per concentration of EOAM and 10 for the control). Insect rearing was carried out until all females died [29].

### 4.5. EOAM Phytotoxicity Bioassay on A. melanocarpa

Experiments on the reaction of black chokeberry to the tested solutions were carried out under laboratory conditions. To assess the phytotoxicity, 15 cm stem sections were used. A triple application of EOAM solutions was carried out using an atomizer (100 mL). The plants were inspected for phytotoxicity symptoms 48 and 72 h post-application. When phytotoxic effects were observed, the severity was evaluated based on the percentage of leaf surface displaying symptoms, such as necrotic spots or discoloration of the leaf blade. The severity was categorized as follows: none (0–1%), slight (1–25%), medium (25–50%), and high (>50%) [78].

### 4.6. Enzymatic Analyses

For biochemical analyses, larvae (L_4_) and black chokeberry inflorescences were placed in plastic 25 × 15 cm insect containers with moistened filter paper to avoid drying [29].

About thirty larvae of *A. advenella* and ten inflorescences were placed in the center at the bottom of each box. One inflorescence consisted of 10 individual buds of black chokeberry. After two hours, larvae and inflorescences were sprayed with an emulsion of EOAM or a control solution that was prepared as described above. The experiments were conducted for 24 and 48 h. Collected insects were homogenized with 3 mL of cold 0.2 M phosphate buffer pH 5.8 for α- and β-glucosidase assay and with 0.2 M phosphate buffer pH 7.0 for CAT and POX or Tris-HCl buffer pH 7.4 for PPO. Acetylcholinesterase (AChE) was extracted from the insect tissues by homogenization with 1 cm^3^ of ice-cold 0.1 M Tris-HCl buffer (pH 7.8) containing 20 mM NaCl and 0.5% Triton X-100. The homogenates were filtered through two layers of cheesecloth and centrifuged at 10,000× *g* for 15 min (centrifuge type Z 323 K, Hermle Labortechnik GmbH, Wehingen, Germany). The supernatants were used in further analyses. All enzymatic analyses were conducted in three independent replicates with the use of a UV–VIS spectrophotometer, type HP 8453 (Hewlett-Packard, Waldbronn, Germany) [29]. All chemical reagents used for enzymatic assays were obtained from Sigma-Aldrich (Poznań, Poland).

CAT activity was measured using Aebi’s [90] method. For this purpose, 0.1 mL of insect homogenate was mixed with 0.1 mL 75 mmol hydrogen peroxide and 0.8 mL extraction buffer. The amount of decomposed H_2_O_2_ after 1 min of enzymatic reaction was measured at 240 nm. The activity of the enzyme was calculated as mM H_2_O_2_ × min^−1^ × mg^−1^ protein.

POX activity was assayed according to Ferman and Dimond [91]. The reaction mixture contained 0.1 mL homogenate of insect tissues, 0.1 mL 0.2 M pyrogallol, 0.5 mL extraction buffer, 0.35 mL distilled water, and 0.05 mL 3% hydrogen peroxide solution. It was incubated at 30 °C for 25 min. The enzymatic reaction was subsequently interrupted by adding 0.25 mL 25% trichloroacetic acid (TCA) solution. The content of purpugallin produced was measured spectrophotometrically at 430 nm. A unit of enzyme activity was defined as mM purpurgallin × min^−1^ × mg^−1^ protein.

PPO activity was determined by the method described by Miles [92] with further modifications [93]. To 0.25 mL of insect homogenate, 0.2 mL of extraction buffer and 0.25 mL of 10 mM catechol solution were added. The changes in absorbance were measured at 460 nm after incubation at 30 °C for 1 h. Enzyme activity was expressed as ΔA × min^−1^ × mg^−1^ protein.

The α- and β-glucosidase activities were assayed according to Katagiri [94] and Chararas and Chipoulet [95]. p-Nitrophenyl-α-D-glucopyranoside (pNαG) and p-nitrophenyl-β-D-glucopyranoside (*p*NβG) were used as substrates for α- and β-glucosidase, respectively. The reaction mixture contained 0.2 mL of insect homogenate, 0.1 mL of phosphate buffer pH 5.8, and 0.2 mL of *p*NαG (15 mM solution in extraction buffer) or pNβG (50 mM solution in extraction buffer). The enzymatic reaction ran for 60 min at 30 °C, and then it was stopped by adding 3 mL of 2% sodium carbonate solution. The content of released p-nitrophenol was measured spectrophotometrically at 405 nm. The activities of both enzymes were expressed as μmol *p*-nitrophenol × hour^−1^ × mg^−1^ protein.

AChE activity was assayed with the use of Ellman et al.’s [96] method. The insect homogenate (0.3 mL) was mixed with 0.4 mL extraction buffer and 0.05 mL of the substrate (75 mM solution of acetylthiocholine iodide—ATChI) and next incubated at 30 °C for 20 min. The enzymatic reaction was stopped by the addition of 0.05 mL 0.644 mM solution of eserine. Then, 0.05 mL of 10 mM 5,5′-dithiobis-2-nitrobenzoic acid (DTNB) solution with 0.018 M of sodium bicarbonate was added to the reaction mixture. The absorbance was measured at 412 nm, and the extinction coefficient (134.6 mM^−1^ × cm^−1^) was used to convert absorbance into molarity. The enzyme activity was expressed as nM ATChI hydrolyzed by mg enzymatic protein during one minute of the reaction (nM min^−1^ × mg^−1^ of protein).

Protein content in all analyzed enzymatic extracts was assayed using the method of Lowry et al. [97].

### 4.7. Statistical Analysis

The results in figures and tables are presented as arithmetic means with standard deviation (SD). The Shapiro–Wilk test was used to assess the normality of data distribution. For the free-choice bioassay, differences between the number of larvae on treated inflorescences and those on the control were analyzed using the Student’s *t*-test. Mortality rates and demographic parameters were analyzed using one-way ANOVA, and differences between means were determined using Tukey’s post hoc test at a significance level of *p* ≤ 0.05. When the data deviated from a normal distribution, the Kruskal–Wallis test was applied as a non-parametric alternative to ANOVA. In such a case, Dunn’s test was used as the post hoc test (for enzyme activities—CAT, POX, PPO, α- and β-glucosidase, and AChE). Probit analysis was conducted to estimate LC_50_ values along with their corresponding 95% confidence limits (CLs) using IBM SPSS v. 23 (IBM Corp., Armonk, NY, USA). All statistical analyses were conducted using Statistica v. 13.0 software (Statsoft, Kraków, Poland).

## 5. Conclusions

The present study is the first report of the insecticidal activity of EOAM against *A. advenella*. The highest tested concentration of EOAM resulted in the strongest insecticidal effect against *A. advenella* larvae and adversely affected the pupation process. The application of EOAM to larvae was associated with increased activity of most of the enzymes analyzed. However, the observed increase in AChE activity in *A. advenella* larvae may indicate a high tolerance of this species to the tested substances. To enhance the efficacy of EOAM, future research should focus on testing higher concentrations of EOAM and consider the different larval growth stages, as individuals in the early developmental stages exhibit increased sensitivity to biologically active compounds. Future studies should also explore synergistic effects between EOAM components and assess the response of *A. advenella* to individual constituents. This will provide a deeper understanding of EOAM’s potential in managing *A. advenella*, a major pest in black chokeberry crops.

## Figures and Tables

**Table 1:** The composition of the essential oil of *Achillea millefolium* (Nanga, Złotów, Poland).

No.	Compound	Retention Time [min]	CAS no.	Retention Index	Quantity [%]
1.	α-Thujene	4.971	2867-05-2	928	0.06
2.	α-Pinene	5.213	7785-70-8	934	1.87
3.	Camphene	5.507	79-92-5	948	0.33
4.	Sabinen	6.065	3387-41-5	974	2.28
5.	β-Pinene	6.177	127-91-3	979	12.84
6.	β-Myrcene	6.700	123-35-3	994	0.22
7.	2-Carene	7.291	4497-92-1	1017	0.16
8.	β-Cymene	7.485	535-77-3	1026	0.54
9.	Eucalyptol	7.593	470-82-6	1031	9.15
10.	γ-Terpinene	8.389	99-85-4	1059	1.29
11.	Linalool	9.217	78-70-6	1102	0.40
12.	α-Campholenal	9.668	4501-58-0	1130	0.30
13.	*trans*-Pinocarveol	10.535	1674-08-4	1141	0.21
14.	Camphor	10.632	76-22-2	1146	2.74
15.	Menthone	10.989	14073-97-3	1154	0.34
16.	Isoborneol	11.188	124-76-5	1156	0.44
17.	Pinocarvone	11.288	30460-92-5	1165	2.88
18.	Terpinen-4-ol	11.624	20126-76-5	1179	2.35
19.	α-Terpineol	12.020	10482-56-1	1194	2.39
20.	*trans*-p-Menth-1-en-3-ol	12.621	16721-39-4	1214	0.08
21.	Piperitone	13.776	89-81-6	1259	0.20
22.	4-Thujen-2-α-yl acetate	14.370	95875-05-1	1274	0.08
23.	Bornyl acetate	14.572	5655-61-8	1288	1.45
24.	Lavandulyl acetate	14.869	25905-14-0	1292	0.12
25.	Thymol	15.064	89-83-8	1299	0.06
26.	Eugenol	15.252	97-53-0	1363	0.20
27.	Capric acid methyl ester	15.777	110-42-9	1371	0.08
28.	α-Ylangene	16.283	14912-44-8	1374	0.14
29.	Copaene	16.930	3856-25-5	1377	0.31
30.	β-Bourbonene	17.147	5208-59-3	1385	0.31
31.	β-Longipinene	17.363	39703-25-8	1415	0.38
32.	β-Caryophyllene	18.015	87-44-5	1422	7.26
33.	β-Copaene	18.249	18252-44-3	1431	1.02
34.	Isogermacrene D	18.633	317819-80-0	1483	0.45
35.	γ-Humulene	18.837	6753-98-6	1484	1.39
36.	Isocaryophillene	19.028	118-65-0	1485	0.31
37.	γ-Amorphene	19.436	6980-46-7	1486	0.70
38.	γ-Curcumene	19.541	28976-68-3	1487	0.58
39.	Ar-Curcumene	19.625	644-30-4	1489	0.85
40.	*epi*-Cubebol	19.925	38230-60-3	1498	0.71
41.	Guaia-6,9-diene	20.016	36577-33-0	1442	0.29
42.	α-Muurolene	20.333	10208-80-7	1503	0.71
43.	Δ-Cadinene	20.561	483-76-1	1527	1.42
44.	Alloaromadendrene oxide-(2)	20.885	85710-39-0	1536	0.23
45.	α-Calacorene	21.013	21391-99-1	1548	0.12
46.	Unknown	21.223	−	1554	0.28
47.	Unknown	21.304	−	1562	0.16
48.	Nerolidol	21.570	2306-78-7	1567	3.79
49.	Spathulenol	21.821	77171-55-2	1584	1.23
50.	Caryophyllene oxide	21.920	1139-30-6	1589	4.47
51.	Viridiflorol	22.140	552-02-3	1598	1.54
52.	Humulene epoxide II	22.426	19888-34-7	1615	0.76
53.	γ-Eudesmol	22.517	473-15-4	1629	0.40
54.	Unknown	22.617	−	1634	0.40
55.	Unknown	22.991	−	1640	0.72
56.	Caryophylla-4(12),8(13)-dien-5α-ol	23.150	19431-79-9	1643	0.73
57.	τ-Cadinol	23.259	5937-11-1	1647	0.66
58.	α-Cadinol	23.554	481-34-5	1662	2.23
59.	Unknown	23.944	−	1679	1.68
60.	Aromadendrane-4,10-diol	24.120	70051-38-6	1691	0.27
61.	Eudesma-4(15),7-dien-1β-ol	24.255	119120-23-9	1711	1.32
62.	Unknown	24.959	−	1736	0.30
63.	Chamazulene	25.205	529-05-5	1741	9.05
64.	Bisabolone	25.578	72441-71-5	1742	0.48
65.	α-Springene	25.909	77898-97-6	1781	0.32
66.	Hexahydrofarnesyl acetone	27.614	502-69-2	1830	1.58
67.	Hexadecanoic acid, methyl ester	29.229	112-39-0	1927	1.97
68.	Phytol	30.336	150-86-7	1951	0.22
69.	Unknown	32.342	−	2057	1.82
70.	9,12-Octadecadienoic acid, ethyl ester	32.342	112-63-0	2078	1.25
71.	18-Octadec-9-enolide	32.697	−	2099	0.56
72.	Linolenic acid methyl ester	33.148	463-40-1	2105	0.49
73.	Unknown	33.665	−	2125	0.88
74.	Unknown	34.609	−	2159	0.20
Oil yield (%)					0.31

The percentage of the analyte was determined based on the peak normalization method.

**Table 2:** Settling inhibition activity (SI) of EOAM toward *Acrobasis advenella* larvae.

Concentration (%)	No. of Larvae Per Inflorescence ± SD		*p*	SI (%)
	Treated	Control		
0.5	6.3 ± 0.577	5.6 ± 0.577	0.23	37.5
0.8	4.0 ± 1.0	7.33 ± 1.155	0.0194	45.3
1.0	4.33 ± 0.577	7.00 ± 1.00	0.016	38.6

Numbers represent the mean number (±SD) of larvae that settled on the treated and control inflorescences; *p* ≤ 0.05 denotes statistically significant differences (Student *t*-test).

**Table 3:** Toxicity (LC_50_) of the EOAM to *A. advenella* larvae after 24, 48, and 72 h of exposure time.

Exposure Time (In Hours)	LC_50_ ^a^	95% CL ^b^	Regression Curve ± SE	χ^2^	*p*
			Slope		
24	1.85	1.38–3.70	0.923 ± 0.288	19.11	0.385
48	1.22	1.07–1.49	1.507 ± 0.256	12.29	0.832
72	0.99	0.88–1.15	1.542 ± 0.222	10.74	0.905

a—lethal concentration (% *w*/*v*)*;* b—confidence limit, which has been calculated with 95% confidence.

**Table 4:** Concentration effect of EOAM on demographic parameters of *Acrobasis advenella*.

EOAM Concentration (%)	Pupa	Adults				Female Fertility		
	Pupation (%) ± SD	Emergence (%) ± SD	Longevity (Days)					
			Min.	Max.	Mean ± SD	Min.	Max.	Mean ± SD
0.5	35.56 ± 3.85 b	75.57 ± 21.42 a	5	11	8.20 ± 1.620 a	87	149	118.30 ± 20.47 a
0.8	22.22 ± 13.88 b	86.67 ± 23.09 a	5	10	7.50 ± 1.65 a	65	156	115.60 ± 27.35 a
1.0	15.55 ± 3.85 b	88.90 ± 19.23 a	5	11	9.00 ± 1.41 a	16	153	107.10 ± 44.38 a
Control	93.33± 6.67 a	100.00 ± 0 a	5	12	8.00 ± 1.83 a	107	150	128.20 ± 13.55 a
	F_(1,3)_ = 56.5156 *p* = 0.000010	F_(1,3)_ = 0.8841 *p* = 0.489306			F_(1,3)_ = 0.4462 *p* = 0.721488			F_(1,3)_ = 0.9196 *p* = 0.441190

The data in the table are mean ± SD (standard deviation) of six replicates; means followed by different letters in columns are significantly different at *p* ≤ 0.05 (Tukey’s HSD test).

**Table 5:** Phytotoxicity of EOAM on *A. melanocarpa* leaves.

Exposure Time (In Hours)	Concentration of EOAM (% *w*/*v*)		
	0.5	0.8	1.0
48	−	−	−
72	−	−	−

Evaluation of phytotoxicity severity was assessed as leaf surface percentage with symptoms: none “−” (0–1%).

## Data Availability

The data that support the findings of this study are available from the corresponding author upon reasonable request.

## Supplementary Materials

The following supporting information can be downloaded at: https://www.mdpi.com/article/10.3390/molecules30091927/s1, Figures S1–S7: MS spectra for unidentified substances of EOAM.
