# Supplementary material for: Chemical Composition and Toxicity of Achillea millefolium L. Essential Oil Against Acrobasis advenella (Lepidoptera, Pyralidae) Under Laboratory Conditions
Source: Molecules. 2025 Apr 26;30(9):1927. doi: 10.3390/molecules30091927 (PMC12073657; doi:10.3390/molecules30091927)
Supplement: Supplementary file 1 [file molecules-30-01927-s001.zip › molecules-3540599-supplementary.pdf]

Figures S1–S7. MS spectra for unidentified substances:

Compound #46

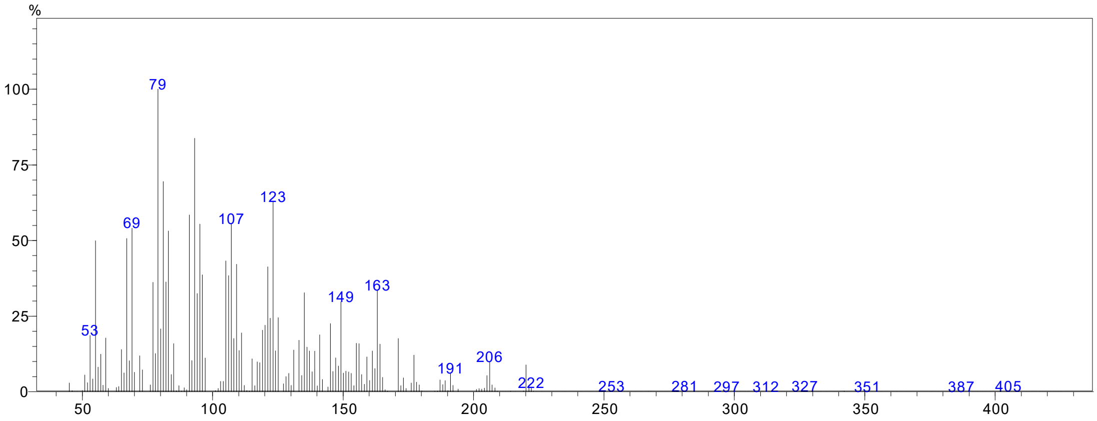

Compound #47

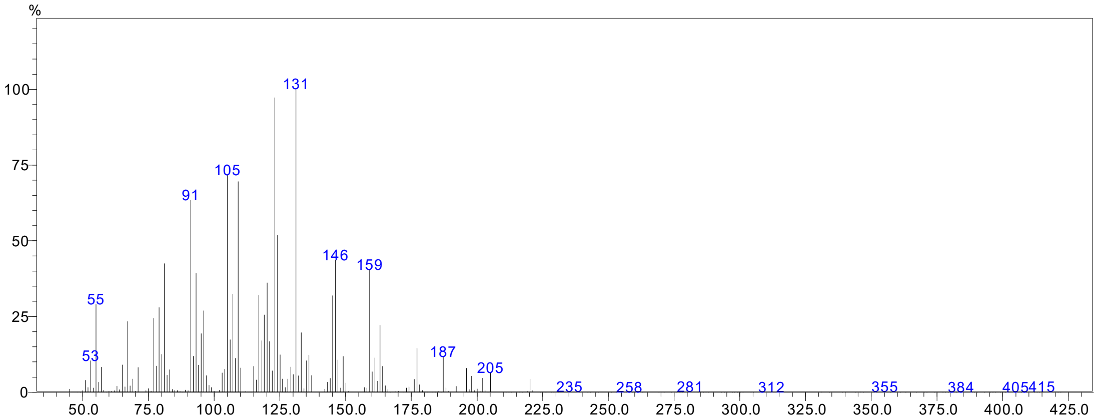

Compound #54

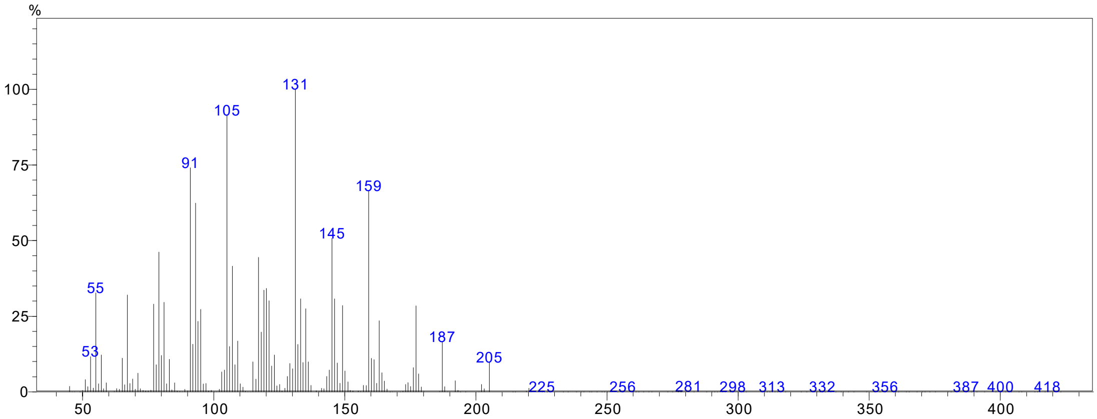

Compound #59

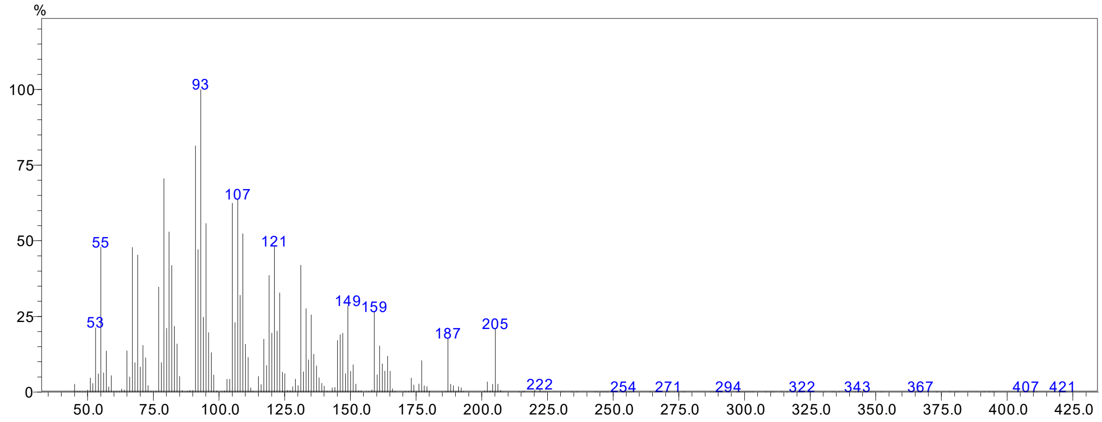

Compound #62

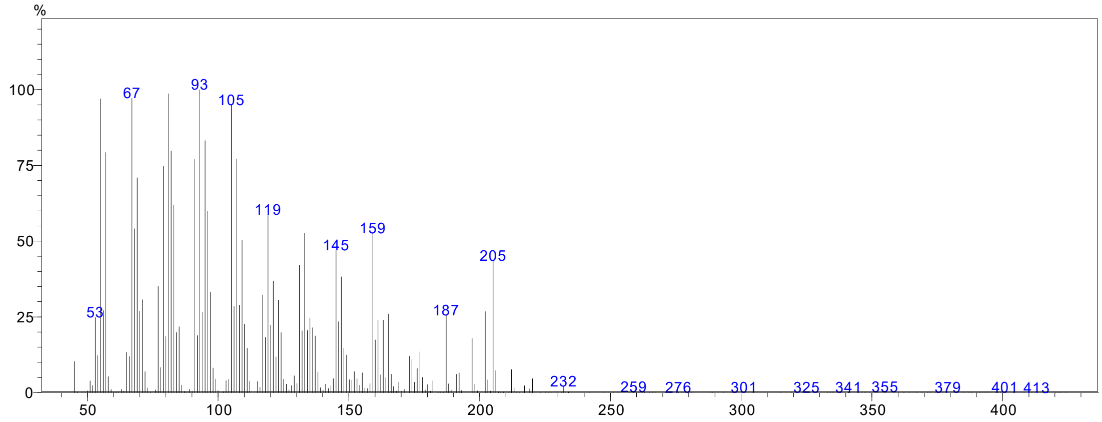

Compound #65

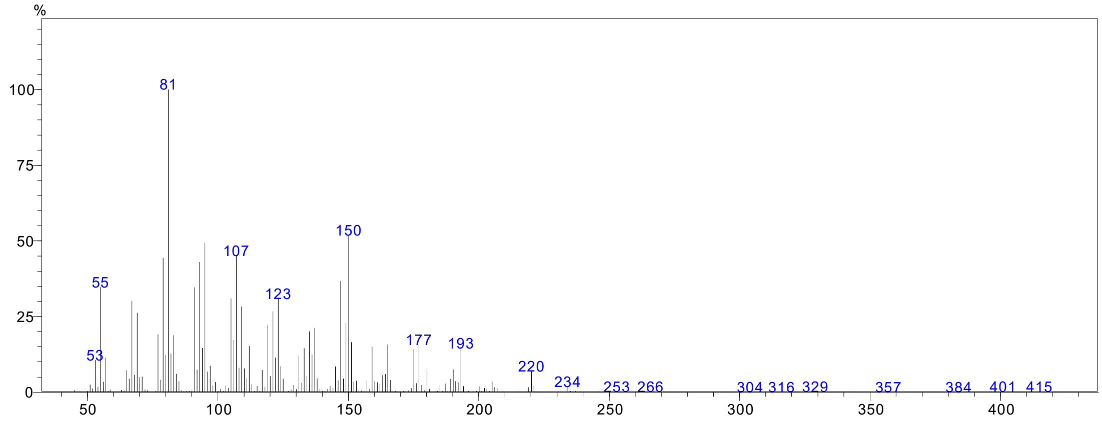

Compound #74

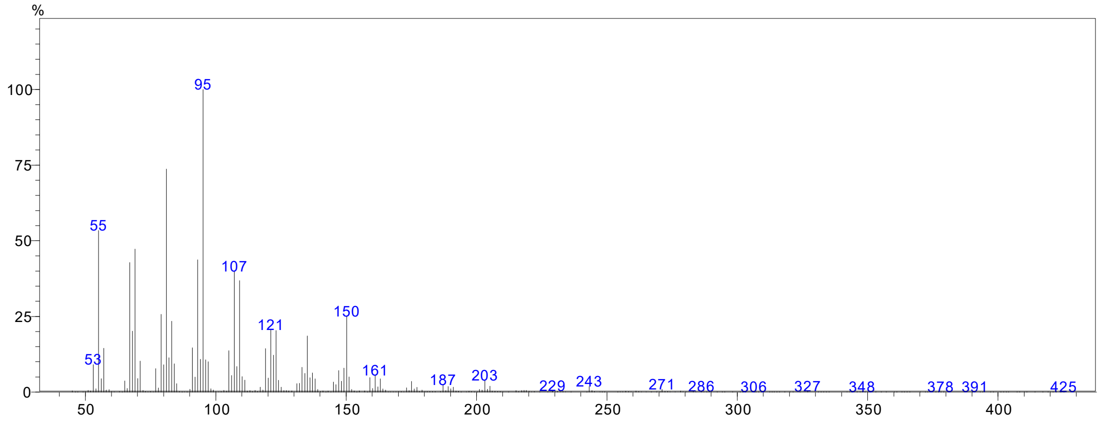

%
100
75
50
25
0
95
55
107
150
121
53
161
187
203
229
243
271
286
306
327
348
378
391
425
50
100
150
200
250
300
350
400
